# Ethnic Disparities in Treatment of Chronic Pain in Individuals with Parkinson's Disease Living in the United Kingdom

**DOI:** 10.1002/mdc3.13430

**Published:** 2022-03-09

**Authors:** Katarina Rukavina, Josephine Ocloo, Magdalena Krbot Skoric, Anna Sauerbier, Omotola Thomas, Juliet Staunton, Olabisi Awogbemila, Dhaval Trivedi, Alexandra Rizos, K. Ray Chaudhuri, King's Parkinson's Disease Pain Scale Group

**Affiliations:** ^1^ Department of Basic and Clinical Neuroscience The Maurice Wohl Clinical Neuroscience Institute, Institute of Psychiatry, Psychology & Neuroscience (IoPPN) at King's College London and King's College Hospital NHS Foundation Trust London UK; ^2^ Parkinson's Foundation Centre of Excellence King's College Hospital London UK; ^3^ Centre for Implementation Science, Health Services, Population and Research Department Institute of Psychiatry, Psychology & Neuroscience (IoPPN), King's College London London UK; ^4^ National Institute for Health Research (NIHR) Applied Research Collaboration South London (NIHR ARC South London) At King's College Hospital NHS Foundation Trust London UK; ^5^ Laboratory for Cognitive and Experimental Neurophysiology, Department of Neurology University Hospital Centre Zagreb Zagreb Croatia; ^6^ Department of Neurology University Hospital Cologne Cologne Germany; ^7^ World Parkinson Coalition New York New York USA; ^8^ Parkinson's Africa UK

**Keywords:** Parkinson's disease, pain, ethnic disparities, appropriate analgesia

## Abstract

**Background:**

Over 80% people with Parkinson's disease (PD; PwP) live with chronic pain.

**Objective:**

Whether ethnic disparities in receipt of appropriate analgesia exist among PwP with chronic pain living in the United Kingdom (UK).

**Methods:**

A retrospective datamining of an existing King's PD Pain Questionnaire validation study dataset enrolling 300 PwP.

**Results:**

69 PwP: 23 Black (57% female), 23 Asian (57% female) and 23 White (65% female) had similar pain burden on the King's PD Pain Scale. Significantly more White PwP (83%) received pain relief compared to Black (48%) and Asian (43%) PwP (*p* = 0.016). The difference was most evident for opioid analgesics (White 43% vs. Black 4% vs. Asian 4%, *p* ≤ 0.001).

**Conclusions:**

Ethnic disparities in the analgesic use among PwP with chronic pain living in the UK are evident in this retrospective analysis, prompting large‐scale studies and reinforcement of interventions to tackle the impact ethnicity might have on the successful analgesia.

Chronic pain, present in over 80% People with Parkinson's disease (PD, PwP), is a challenging non‐motor symptom (NMS), adversely affecting the health‐related quality of life.^1^, ^2^, ^3^ In individuals from ethnic minorities, a distinctive non‐motor dominant clinical phenotype of PD, marked by a greater NMS burden, including heightened pain experience, has been reported.^4^

Disparities in diagnosis, treatment and survival of PwP from ethnic minorities, compared to White PD patients, exist.^5^ Studies suggest that former are more likely to self‐report less disability relative to clinician‐observed motor impairment and are at higher risk of delayed or missed PD diagnosis, receive health‐care more frequently in emergency departments or as an inpatient (rather than in an outpatient setting) and have worse survival rate.^5^, ^6^ Furthermore, PwP from ethnic minorities are significantly less likely to receive at least one dopamine replacing therapy, pharmacological treatment for affective disorders or physiotherapy.^5^, ^7^ Of note, these studies mainly originated from the United States, while limited evidence is available from the United Kingdom (UK).^4^

Similarly, considerable ethnic health‐care disparities in receipt of appropriate pain relief have been reported in a range of various painful conditions globally; whether such disparities exist among individuals with PD and chronic pain living in the UK is unknown.

We conducted a retrospective analysis of the existing database collected as a part of the King's PD Pain Questionnaire (KPPQuest) validation study and explored the patterns of analgesics use among PwP with chronic pain from the three main ethnic groups living in the UK: Asian, Black and White.^8^

## Methods

We performed a retrospective case–control analysis of patients enrolled in the KPPQuest validation study (carried out between August 2013 and February 2016).^8^ In this study, written informed consent was obtained from all participants. The original study was authorized by local ethics committee (NRES North East—Newcastle & North Tyneside REC2, 105,735, 12/NE/0365) and adopted as a national study by the National Institute of Health Research Clinical Research Network in the UK (UKCRN No. 13344).^8^

The ethnicity was self‐reported based on the 18 categories recommended by the Office for National Statistics, reduced down to four groups: White (White—British, White—Irish, any other White), Black (Black—African, Black—Caribbean, any other Black), Asian (Indian, Pakistani, Bangladeshi, Chinese, any other Asian), and Mixed/Other.^9^ Patients with missing ethnicity data were excluded.^10^

Socio‐demographic characteristics (age, sex), PD history (disease duration, Levodopa Equivalent Daily Dose (LEDD; calculated according to Tomlinson)) and analgesics consumption were noted from the database.^11^ Analgesics were classified into three categories: non‐steroidal anti‐inflammatory drugs (NSAIDs), opioid analgesics and anticonvulsants.

The following assessments were used:

1. *King*'*s Parkinson*'*s Disease Pain Scale (KPPS)*, a rater‐interview‐based scale, was used to identify and quantify pain experienced by PD patients in the past month and was the main anchor in this study. Each of the 14 items of the KPPS focuses on a specific type of pain and rates its severity and frequency. The items are grouped into seven domains: musculoskeletal, chronic, fluctuation‐related, nocturnal, oro‐facial, discoloration and oedema/swelling, and radicular pain.^12^ 2. *Hoehn and Yahr (HY)* stage.^13^ 3. *Parkinson*'*s Disease Questionnaire 8 (PDQ‐8)*, **eight‐item patient‐completed questionnaire**, was used to quantify self‐perceived health.^14^ 4. *Non‐motor Symptoms Scale (NMSS)* is a 30‐item, health‐care professional completed tool for assessment of non‐motor burden. *Domain 2* was used to assess the severity of sleep disturbances, and *the Domain 5* for self‐perceived cognitive complaints.^15^ 5. *Hospital Anxiety and Depression Scale (HADS)*, a brief self‐assessment mood scale, was used to assess presence of clinically significant degrees of anxiety and depression.^16^

### Statistical Analysis

Statistical analysis was carried out using SPSS Statistics software, version 26 (IBM, Armonk, NY, USA). All Black and Asian participants were identified from the dataset. Next, an equal number of White study participants was selected based on the nearest neighbor matching (at the ratio 1:1) performed by a researcher blinded for analgesics acquisition, using sex and the closest age, HY stage, and KPPS total score to form three groups with the same sample size. Descriptive statistics were provided for continuous and categorical variables. One‐sample Kolmogorov–Smirnov test was used to estimate whether the data in the sample were normally distributed. For certain variables with non‐parametric distribution, results were expressed as median (range)/mean ± standard deviation to aid clinical understanding. The differences between the ethnic groups were analyzed using ANOVA or Kruskal‐Wallis test, as appropriate. Pearson's chi‐square test was used to analyze between‐group differences in categorical variables, including analgesics use. The observed differences were considered statistically significant if the *p* value was less than .05. Conducting Spearman's correlations, we examined associations between KPPS total score, sleep disturbances (NMSS Domain 2), cognitive complaints (NMSS Domain 5), and HADS scores for anxiety and depression across the three different ethnic groups.

## Results

We identified 69 individuals to be included in our analysis: 23 Black (57% female) and 23 Asian (57% female) PwP, matched with 23 White (65% female) PD patients. Clinical and demographic characteristics are shown in the Table 1; participants were from London region, where over 40% residents identify as ethnic minorities.^9^

**TABLE 1:** Patients’ demographic and clinical characteristics

	White N *= 23*	Black N *= 23*	Asian N *= 23*	*P*
Age (Years; Mean ± SD)	62.43 ± 11.50	62.00 ± 11.94	64.52 ± 7.99	0.692
Sex (Female: n, %)	15 (65%)	13 (57%)	13 (57%)	0.863
Hoehn & Yahr stage (Median, Range)	2 (1–4)	2 (1–4)	2 (1–4)	0.151
Disease duration (Years; Median, Range/Mean ± SD)	2 (0–16)/4.26 ± 4.82	3 (0–17)/5.02 ± 4.77	6 (0–30)/7.65 ± 6.18	0.024* Asian vs. White 0.008* Asian vs. Black 0.058 White vs. Black 0.451
LEDD (mg; Mean ± SD)	661.28 ± 450.11	564.02 ± 499.87	765.92 ± 441.48	0.343
KPPS total score (Median, Range/Mean ± SD)	16 (2–74)/21.7 ± 18.92	16 (2–72)/21.65 ± 18.87	20 (3–76)/26.61 ± 20.06	0.425
NMSS Domain 5 score (Median, Range/ Mean ± SD)	1 (0–24)/5.35 ± 7.75	0 (0–14)/1.87 ± 3.46	3 (0–28)/7.57 ± 9.66	0.054
NMSS Domain 2 score (Median, Range/Mean ± SD)	10 (0–38)/13.48 ± 12.14	14 (0–27)/12.00 ± 7.47	16 (0–38)/16.00 ± 11.56	0.438
HADS Anxiety Score (Mean ± SD)	6.96 ± 4.34	4.74 ± 3.48	6.74 ± 4.74	0.141
HADS Depression Score (Mean ± SD)	6.61 ± 4.27	5 ± 2.88	7.35 ± 4.11	0.109
Pain medication (n, %)	19 (83%)	11 (48%)	10 (43%)	0.016*
NSAID, Paracetamol (n, %)	10 (43%)	10 (43%)	10 (43%)	1.000
Opioids (n, %)	10 (43%)	1 (4%)	1 (4%)	≤0.001*
Anticonvulsants (n, %)	1 (4%)	0 (0%)	1 (4%)	1.000
More than one pain medication (n, %)	2 (8%)	0 (0%)	4 (17%)	0.153
PDQ‐8 SI (Mean ± SD)	34.92 ± 22.84	30.30 ± 17.66	39.40 ± 22.67	0.352

Abbreviations: HADS, Hospital Anxiety and Depression Scale; KPPS, King's Parkinson's Disease Pain Scale; LEDD, Levodopa Equivalent Daily Dose; NSAID, non‐steroidal anti‐inflammatory drugs; NMSS, Non‐motor Symptoms Scale; PDQ‐8, PD Questionnaire 8; SD, standard deviation.

*statistically significant difference, *P* ≤ 0.05.

There were no statistically significant differences in the total burden of pain, as estimated using KPPS, between the three groups. Table 1. The scores on the individual domains of the KPPS, labelling the severity of distinctive sub‐types of the PD‐related pain, did not differ significantly across the groups.

**FIG. 1:**
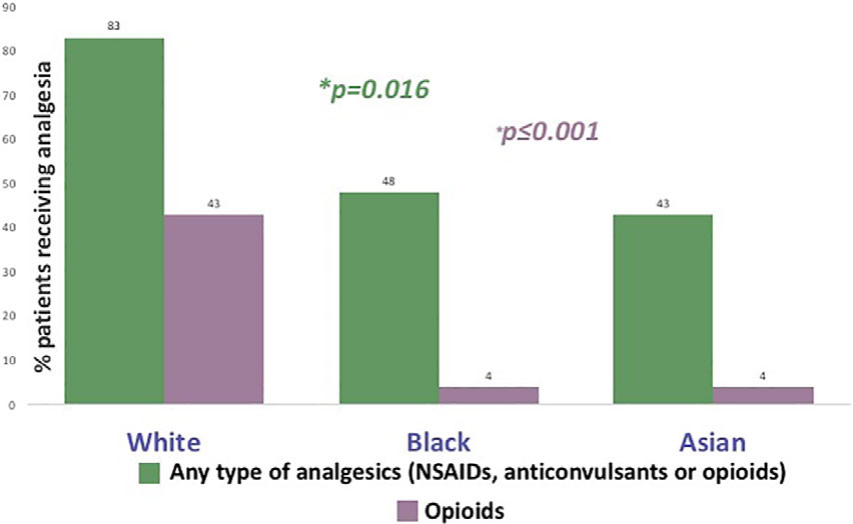
A proportion of patients receiving pain medication in different ethnic groups. *statistically significant difference, *p* ≤ 0.05.

Overall, higher proportion of White PwP (83%) used pain medication compared to Black (48%) and Asian (43%) ethnic groups (*p* = 0.016). In particular, the use of opioid analgesics was significantly higher among White PwP (White 43% vs. Black 4% vs. Asian 4%, *p* ≤ 0.001). The usage of NSAIDs (White 43% vs. Black 43% vs. Asian 43%, *p* = 1.000) and anticonvulsants (White 4% vs. Black 0% vs. Asian 4%, *p* = 1.000), or the proportion of patients receiving more than one pain medication (White 8% vs. Black 0% vs. Asian 17%, *p* = 0.153) did not differ significantly between the three groups. Fig. 1.

No statistically significant differences in the quality of life (as declared on PDQ‐8), anxiety or depression (HADS scores for depression and for anxiety), severity of sleep disturbances (NMSS, Domain 2) and self‐perceived cognitive complaints (NMSS, Domain 5) were found across the different ethnic groups. Table 1.

However, in White PwP, the KPPS total score showed statistically significant positive correlation with sleep disturbances (r_s_ = 0.624, *p* = 0.001), cognitive scores (r_s_ = 0.538, *p* = 0.008), depression (r_s_ = 0.666, *p* = 0.001) and anxiety (r_s_ = 0.457, *p* = 0.028), whereas, in Black and Asian PwP, no statistically significant associations were found between burden of pain and cognitive complaints (Black: *p* = 0.656, Asian: *p* = 0.705), anxiety (Black: *p* = 0.318, Asian: *p* = 0.686), or depression (Black: *p* = 0.971, Asian: *p* = 0.255). Sleep disturbances significantly correlated with pain in Asian (r_s_ = 0.440, *p* = 0.036), but not in Black (*p* = 0.357) PwP.

## Discussion

Despite the similar pain ratings on the KPPS (a scale validated worldwide specifically for assessment of distinctive sub‐types of PD‐related pain), PwP from ethnic minorities received significantly less analgesics than White PwP. Differences were particularly evident in the opioid prescribing, which was higher in the White PwP compared to those from ethnic minorities, whereas the amounts of self‐administered analgesics (eg, NSAIDs) used did not differ between the three ethnic groups.

Our findings suggest that disparities in pain medication, and particularly opioids, prescribing exist in the UK between White, Black and Asian PwP with chronic pain. We can only speculate about the potential causes, which are likely to be complex and multifaceted, and prompt further investigations.

Ethnic disparities in receipt of appropriate pain management appear to be widespread.^17^, ^18^ For example, women from ethnic minorities groups are less likely to receive appropriate analgesia for peripartum pain.^19^, ^20^, ^21^ In general, individuals from ethnic minorities are less likely to receive analgesics for painful conditions with equivocal findings (eg, migraine, back pain).^17^ This is of a particular concern in PD, where chronic pain is highly prevalent, yet often remains undeclared by patients and neglected by clinicians, leading to suboptimal management in many.^22^, ^23^

From an author OT's (a Black person living with PD) perspective, some patients from ethnic minorities, such as herself, may under‐report their pain as they do not feel it is a “serious enough” issue to report until it becomes unbearable. However, for an illness where up to 80% could be living with pain, she believes that health‐care professionals should make a deliberate effort to ensure their patients from ethnic minorities understand that chronic pain is not a condition they have to live with—it can and should be properly managed and must be flagged up in consultations. Our findings support OT's lived experience, pointing towards risk of inadequate pain control in PwP from ethnic minorities.

Of note, some studies pointed out that, even when the access is equal, patients from ethnic minorities tend to use lower than prescribed amounts of analgesics.^24^ One of the possible explanations might be that White patients are more likely to expect immediate pain relief, and thus request and use analgesics more often, while patients from the ethnic minorities are less likely to perceive control over pain and its treatment.^25^, ^26^, ^27^ Nonetheless, the observed differences in receipt of pain relief may be derived, at least partly, from an ethnic bias in perceptions of others' pain—for example, it has been reported that a substantial number of White medical students and residents might hold false beliefs about biological differences between White and Black patients. Assuming a priori that later feel less pain, they might shape insufficient treatment recommendations.^28^

Notably, only one White and one Asian PD patient received an anticonvulsant for pain relief. According to the UK Parkinson's Pain Study (enrolling 1957 patients with early‐to‐moderate PD), up to 33% of PwP might exhibit an alteration of sensory processing.^29^ In those patients, an anticonvulsant would be the most appropriate therapeutic approach.^30^ However, in our study, no conclusion can be drawn concerning the possible causes for the observed under‐use of the anticonvulsants.

Finally, it is generally accepted that sleep disturbances, cognitive impairment, anxiety and depression can aggravate chronic PD‐related pain.^1^, ^2^, ^31^ However, in our study, the link between the cognitive complaints, anxiety or depression and the burden of pain was evident only in White PwP, but not in Asian or Black PwP. In addition, while there was a strong relationship of the severity of sleep disturbances and the burden of pain in White PwP and a moderate one in Asian PwP, no such relationship was found in Black PwP.

In conclusion, disparities in the receipt of pain‐relieving medication may exist between White, Black and Asian PwP with chronic pain living in the UK, as evident in this small retrospective data analysis. High‐quality, large‐scale studies are urgently needed to tackle the impact ethnicity might have on the effective pain management (including both pharmacological and non‐pharmacological strategies for pain relief) and to determine whether patients' outcomes are affected by the observed dissimilarities. This will ultimately allow for the future development of targeted interventions aiming to diminish the potential barriers for successful pain relief in individuals with PD from the ethnic minorities living with chronic pain.

## Author Roles

1. Research Project: A. Conception, B. Organization, C. Execution; 2. Statistical analysis: A. Design, B. Execution, C. Review and critique. 3. Manuscript preparation: A. Writing of the first draft. B. Review and critique.

KR: 1A, 1B, 1C, 2A, 2B, 2C, 3A, 3B

JO: 3B

MKS: 2A, 2B, 2C, 3B

AS: 3B

OT: 3B

JS: 1C, 3B

OA: 3B

DT: 3B

AR: 1C, 3B

KRC: 1A, 1B, 1C, 2C, 3B

## Disclosures

**Ethical Compliance Statement:** The KPPQuest validation study was authorized by local ethics committees (NRES North East—Newcastle & North Tyneside REC2, 105,735, 12/NE/0365) and written informed consent was obtained from all participants. We confirm that we have read the Journal's position on issues involved in ethical publication and affirm that this work is consistent with those guidelines.

**Funding Sources and Conflicts of Interest:** This manuscript presents independent research funded by the National Institute for Health Research (NIHR) Mental Health Biomedical Research Centre and Dementia Unit at South London and Maudsley NHS Foundation Trust and King's College London. The views expressed are those of the author(s) and not necessarily those of the NHS, the NIHR or the Department of Health. This research did not receive any specific grant from funding agencies in the public, commercial, or not‐for‐profit sectors. The authors declare that there are no conflicts of interest relevant to this work.

**Financial Disclosures for the Previous 12 Months:** The authors declare that there are no additional disclosures to report.
